# Structural Features of the αβTCR Mechanotransduction Apparatus That Promote pMHC Discrimination

**DOI:** 10.3389/fimmu.2015.00441

**Published:** 2015-09-03

**Authors:** Kristine N. Brazin, Robert J. Mallis, Dibyendu Kumar Das, Yinnian Feng, Wonmuk Hwang, Jia-huai Wang, Gerhard Wagner, Matthew J. Lang, Ellis L. Reinherz

**Affiliations:** ^1^Laboratory of Immunobiology, Department of Medical Oncology, Dana-Farber Cancer Institute, Boston, MA, USA; ^2^Department of Medicine, Harvard Medical School, Boston, MA, USA; ^3^Department of Biological Chemistry and Molecular Pharmacology, Harvard Medical School, Boston, MA, USA; ^4^Department of Chemical and Biomolecular Engineering, Vanderbilt University, Nashville, TN, USA; ^5^Department of Biomedical Engineering, Texas A&M University, College Station, TX, USA; ^6^Department of Materials Science and Engineering, Texas A&M University, College Station, TX, USA; ^7^School of Computational Sciences, Korea Institute for Advanced Study, Seoul, South Korea; ^8^Department of Pediatrics, Harvard Medical School, Boston, MA, USA; ^9^Department of Molecular Physiology and Biophysics, Vanderbilt University, Nashville, TN, USA

**Keywords:** T cell receptor, antigen recognition, mechanosensor, catch bond, kinetic proofreading, pre-TCR, thymic development, CD3

## Abstract

The αβTCR was recently revealed to function as a mechanoreceptor. That is, it leverages mechanical energy generated during immune surveillance and at the immunological synapse to drive biochemical signaling following ligation by a specific foreign peptide–MHC complex (pMHC). Here, we review the structural features that optimize this transmembrane (TM) receptor for mechanotransduction. Specialized adaptations include (1) the CβFG loop region positioned between Vβ and Cβ domains that allosterically gates both dynamic T cell receptor (TCR)–pMHC bond formation and lifetime; (2) the rigid super β-sheet amalgams of heterodimeric CD3εγ and CD3εδ ectodomain components of the αβTCR complex; (3) the αβTCR subunit connecting peptides linking the extracellular and TM segments, particularly the oxidized CxxC motif in each CD3 heterodimeric subunit that facilitates force transfer through the TM segments and surrounding lipid, impacting cytoplasmic tail conformation; and (4) quaternary changes in the αβTCR complex that accompany pMHC ligation under load. How bioforces foster specific αβTCR-based pMHC discrimination and why dynamic bond formation is a primary basis for kinetic proofreading are discussed. We suggest that the details of the molecular rearrangements of individual αβTCR subunit components can be analyzed utilizing a combination of structural biology, single-molecule FRET, optical tweezers, and nanobiology, guided by insightful atomistic molecular dynamic studies. Finally, we review very recent data showing that the pre-TCR complex employs a similar mechanobiology to that of the αβTCR to interact with self-pMHC ligands, impacting early thymic repertoire selection prior to the CD4^+^CD8^+^ double positive thymocyte stage of development.

## Introduction to αβ T Cell Immunity

Precursors of T (thymus-derived) and B (bone marrow-derived) lymphocytes generate a repertoire of antigen-specific T cell receptors (TCRs) and B cell receptors (BCRs) of immense diversity utilizing somatic rearrangements of variable gene segments (VDJ and VJ recombination) in mammals and other jawed vertebrates. Differentiation and selection processes of those cell types shape two complementary lineages of the immune system, offering protection with exquisite specificity, sensitivity and long-term memory, hallmarks of adaptive immunity.

The mammalian adaptive immune system protects against infectious diseases as well as tumors in a highly specified manner (1). Thymus-derived T lymphocytes detect perturbations among the body’s own cellular surface constituents, distinguishing abnormal from normal cells while B lymphocytes produce secreted antibodies to neutralize circulating toxins and pathogens. Self- vs. non-self-discrimination is an αβ T cell functionality endowed by clonal cell surface TCRs. Within any given mammal, there are millions of distinct αβ T cells each with their own unique αβTCR expressed at 20,000–40,000 copies per T lymphocyte. Whereas antibodies recognize proteins, glycans, viruses, or particulate matter directly, each T lymphocyte identifies a peptide bound to the groove of a major histocompatibility complex (MHC) molecule (HLA in human, H-2 in mouse) displayed as a complex (pMHC) on a nucleated cell. T lymphocytes search for peptides arrayed on the surface of cells in the body as part of immune surveillance, where aberrant processes within a cell may be reflected by alterations of MHC-bound peptides on its surface. Once a T lymphocyte recognizes a variant peptide via its TCR, for example, a foreign peptide derived from a viral proteome bound to a self-MHC molecule, signaling is initiated for cytotoxic T lymphocytes (CTL) to kill such a “flagged” cell. Notwithstanding homeostatic proliferation, mature T cells tend to disregard self-peptide/self-MHC complexes and thus, are inactivation inert. Preoccupation with foreign epitopes bound to self-MHC by αβ T cells is established through screening processes in the thymus. This process employs an apoptotic negative selection mechanism that deletes self-reactive thymocytes, whereas weakly self-reactive thymocytes are nurtured (2).

The exquisite specificity and sensitivity of a high avidity αβ T lymphocyte allow it to recognize several copies of a singular peptide displayed among a sea of unrelated (100,000) but similarly sized moieties (~9 residues in length) on a cell (3). The basis of robust αβTCR recognition has been unclear given the low momoneric affinity of TCR–pMHC interactions in solution. This review shall highlight insights into that paradox. To this end, we will focus on recent discoveries about αβTCR mechanotransduction, dynamic TCR–pMHC bond formation, allostery, and structural transitions linked to TCR complex structure and signaling. Relevance for mature T cell function and thymic repertoire selection are discussed.

## αβTCR Complex Structure: An Overview

The multimeric transmembrane (TM) αβTCR complex is composed of an antigen binding αβ disulfide-linked heterodimer that non-covalently associates with the signal-initiating CD3 subunits (CD3εγ, CD3εδ, and CD3ζζ) [reviewed in Ref. (4–6)]. The CD3ε, γ, and δ subunits, each contain an Ig-like extracellular domain, a short connecting peptide (CP) just proximal to the membrane, a TM domain, and a relatively lengthy cytoplasmic tail. TCR α and β subunits form a variable VαVβ module, which binds pMHC, and a constant region module, which is thought to interact with the CD3 ectodomains. Following an interaction between the T cell surface expressed TCRαβ heterodimer and a pMHC ligand on an antigen-presenting cell (APC), a signaling cascade is initiated via the immunoreceptor tyrosine-based activation motifs (ITAM) in the CD3 cytoplasmic tails (7–9). The CD8 and CD4 co-receptors, on cytotoxic T cells and on helper T cells, respectively, each functions to bring the tyrosine kinase p56lck (lck) to the TCR–pMHC complex for ITAM phosphorylation. The accessibility of the ITAMs to lck phosphorylation is initiated following TCR–pMHC interaction. This, in turn, leads to association of a second tyrosine kinase, ZAP-70, phosphorylation of LAT, PLCγ activation, calcium mobilization (5), and ensuing activation of downstream gene programs (10). The TCR belongs to a group of receptors on immune cells referred to as non-catalytic tyrosine-phosphorylated receptors (11). The NMR and X-ray structures of CD3εγ and CD3εδ reveal parallel pairing of rigidified dimer models (12–15) where the associated TCRαβ heterodimer is itself a rigid structure further enhanced by the β chain constant domain FG loop (16). The functional importance of this contrasting rigidified arrangement of TCR complex dimer components has been suggested by studies, including our own, examining T cell activation via the TCR pMHC ligands under load [Ref. (17–24) and reviewed below].

**Figure 1:**
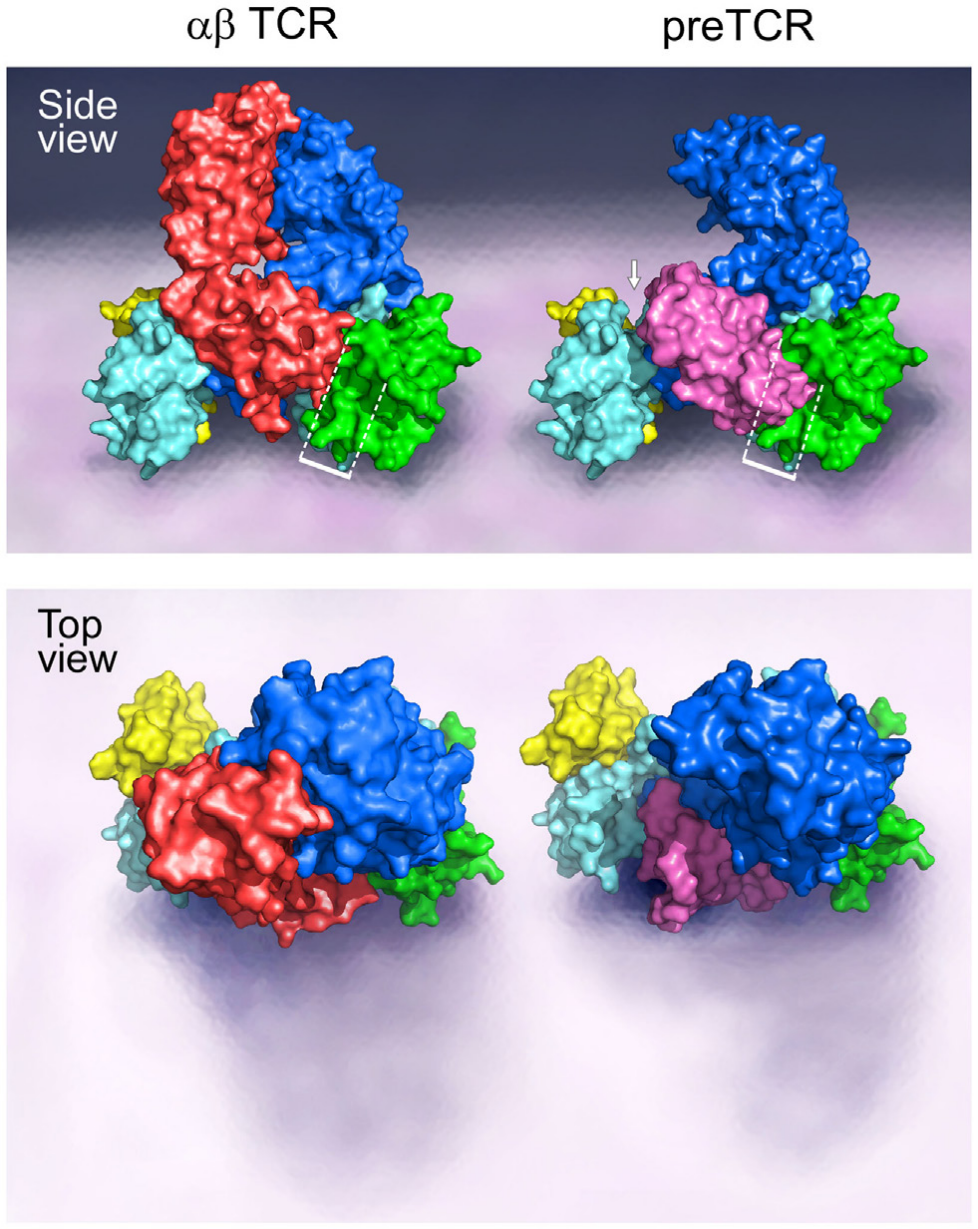
**Models of the αβTCR and the pre-TCR complex**. A side and top view of the assembly of the TCR ectodomain subunits on the surface of a cell membrane are illustrated. The αβTCR model was based on the following PDB codes: 1NFD, 1XMW, and 1JBJ; and the pre-TCR model was based on the PDB code 3OF6. The β chain is shown in blue for both the αβTCR and pre-TCR structures, the α chain is shown in red in the αβTCR and pTα in pink in the pre-TCR structure. For both the αβTCR and pre-TCR, the CD3ε chain is shown in cyan, the CD3γ chain in green, and the CD3δ chain in yellow. In the αβTCR, the CD3εγ heterodimer slots into the Cβ binding cleft in part created by the FG loop, and juxtaposed to the Cα domain. The white bracketed region in the side view illustrates the steric clash of the pTα domain with the CD3εγ heterodimer in this potential CD3 binding cleft. The structural crowding created due to the presence of the pre-TCR pTα chain is suggestive of an altered 3D configuration of the pre-TCR relative to the αβTCR. The white arrow highlights the presence of a significant gap generated between the pTα chain and the CD3εδ heterodimer as a consequence of the missing V domain and structural differences. Such a geometric alteration between the subunits may impose differing signaling requirements on the CD3εδ heterodimer in the pre-TCR vs. the αβTCR. The top view shows the absence of the Vα domain in the pre-TCR with the highly exposed pTα domain and CD3εδ subunits. Note that in the pre-TCR side view, the Vβ hydrophobic patch involves the exposed cell membrane-distal surface (upper left blue surface) buried by Vα in the TCR (34).

A TCR complex structural model, as provided in Figure 1, illustrates the ectodomains of αβ, CD3εγ, and CD3εδ heterodimers, defining one plausible topology among a range of structures. This rendering is based on known αβTCR complex characterization, as detailed previously (15, 16, 25–32). Of note, throughout the text the complete TCR complex (i.e., including the associated CD3 subunits) will be referred to as the αβTCR, whereas the designation TCRαβ refers solely to the heterodimeric component within the complex. The N-linked glycans have been omitted for clarity but are undoubtedly important in regulating movements of the various components of the TCR complex that impact signaling (33). Evident from the αβTCR side view (top) is the TCRαβ heterodimer centrally located within the complex and projecting vertically 80 Å from the cell membrane. The shorter (40 Å) CD3 heterodimers flank either side of the TCRαβ, CD3εδ on the “left” TCRα side and CD3εγ on the “right” TCRβ side. The width of the CD3εδ and CD3εγ subunits, 50 and 55 Å, respectively, are similar in size to the TCRαβ heterodimer (58 Å), and assembled (glycans excluded) span ~160 Å. Lateral tilting motions of the TCRαβ heterodimer upon pMHC binding will likely be hindered due to the presence of the associated CD3 ectodomain components. The VαVβ domain antigen recognition module at the top is relatively flat, well matched for the horizontal pMHC surface (not shown) while the membrane-proximal CαCβ module is similar in height to the CD3 heterodimers. The current model shows the most compact TCR complex structure.

Of note, the pre-T cell receptor (pre-TCR), a pTα-β heterodimer appearing on double negative (DN) CD4^−^CD8^−^ thymocytes before αβTCR expression at the double positive (DP) CD4^+^CD8^+^ stage and associated with the same CD3 heterodimers, must adopt a somewhat different compact topology (Figure 1 side view, right). Overlay of pTα-β on the αβTCR complex by β subunit superposition shows that pTα (pink) impinges upon the CD3εγ ectodomain (dotted rectangle) while requiring repositioning of the CD3εδ heterodimeric ectodomain (arrow). The surface topology of the pre-TCR V module is also distinct, lacking a Vα domain and thus exposing components of Vβ not surface accessible on the TCR VαVβ module. The significance of this difference on pMHC recognition shall be discussed later.

Based on the structures of CD3εδ and CD3εγ, we have hypothesized that highly selective TCR signaling may require dynamic interaction (17) rather than static on-and-off switching. The interface distances between TCRαβ heterodimer and CD3 heterodimers may be small, with their surface contact areas changing during pMHC ligation. Being one of a range of acceptable structures, no detailed information on the interfaces is warranted. Nevertheless, force-dependent structural transitions revealed by single-molecule experiments described below imply that apposition of TCRαβ with CD3εγ and CDεδ subunit heterodimers may change upon pMHC ligation as suggested by recent structural studies (35), impacting downstream signaling. These transformations include creation or ablation of new docking sites, TM conformational changes and accessibility of ITAMs for tyrosine kinase-mediated phosphorylation.

## Molecular Features Facilitating TCR Mechanotransduction

During immune surveillance, T cells scan their environment, physically binding and crawling over structures undergoing cell motility processes that can generate tensile, shear, and compressive stresses over a wide range of forces (piconewton to nanonewton). Additionally, forces within the cell through cytoskeletal (actin, microtubule, etc.) rearrangements can couple to membrane bound structures, such as the TCR complex. Direct evidence that the TCR acts as a mechanosensor was experimentally shown through optical tweezer-based measurements that presented pMHC coated beads to surface bound TCRs, where mere binding without force was insufficient for triggering, but tangential force led to T cell activation (17). The concept of the T cell acting as a mechanosensor may reconcile the discrepancy between the precision in recognition described above and low affinity of free unbound ligand (17, 33, 36, 37). A non-linear response of the TCR–pMHC bond was recently shown in a biomembrane force probe (BFP) assay where single-molecule interactions and signaling can be tracked through repeated measurement (38). Thus, structural aspects of the TCR complex must be adapted to permit discrimination of relevant peptides for immune recognition in an environment that is chemically noisy, given the complexity of similarly sized peptides bound to identical MHC molecules on a cell. In addition, the TCR apparatus must cope with physical noise given various forces generated during cell movement both within and outside of the vasculature and a result of attachment/detachment cycles from other cells and the extracellular matrix. If force is involved in signaling linked to relevant pMHC ligation, then it has to afford a special torque or vector distinct from those other physical forces. Here, we review how structural adaptations of the TCR complex components have evolved within their Ig-like ectodomains and other segments for promoting such specified signaling.

### Structural features of the CβFG loop

**Figure 2:**
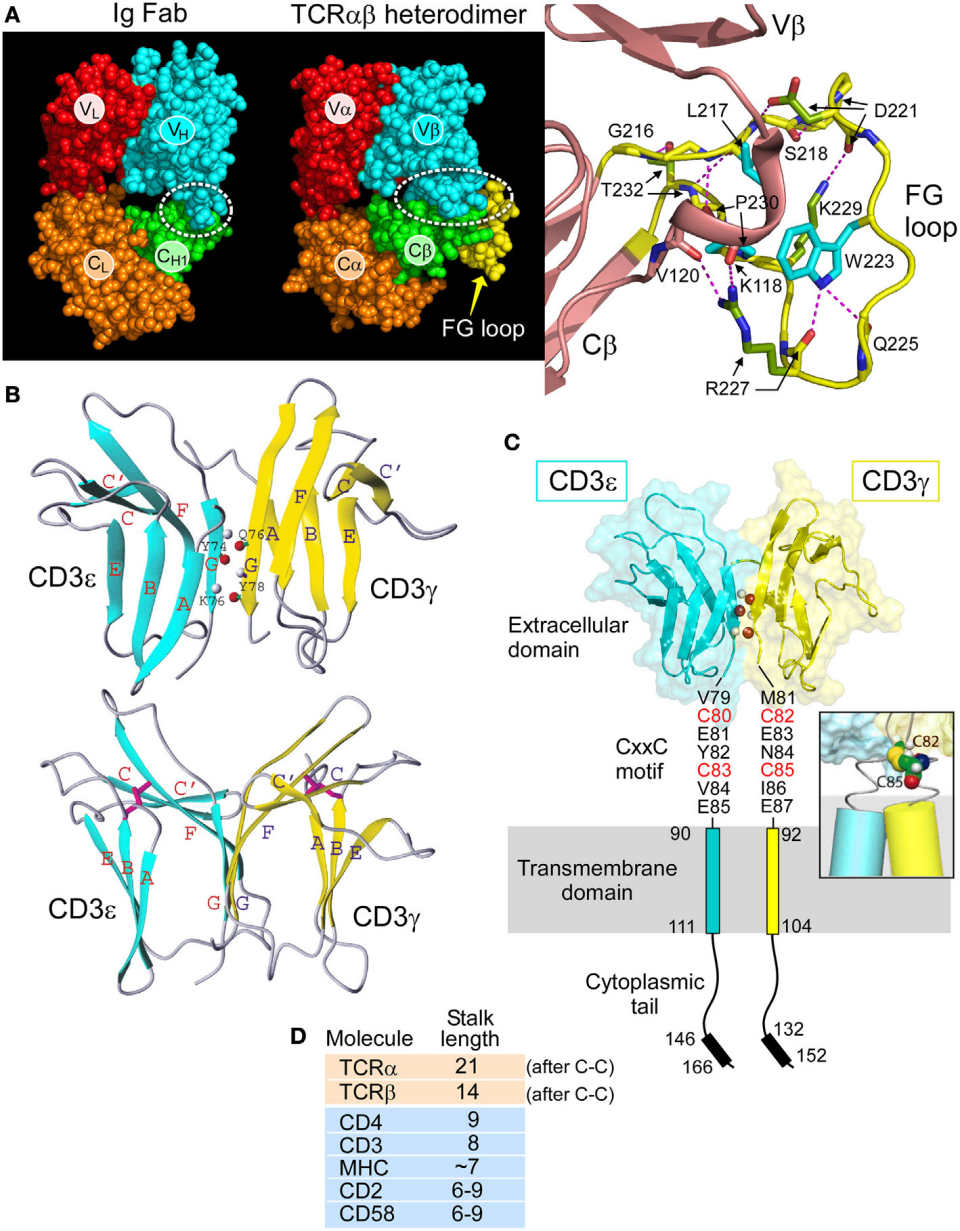
**Rigidifying elements of the TCR**. **(A)** The TCRαβ heterodimer (1OGA) compared with the Ig Fab (4JFX) fragments with equivalent domains represented in their respective CPK format. The dotted ellipse encloses the VβCβ interface. Note the more extensive interface in the TCR. A magnified view of the CβFG loop region is shown in yellow with the three conserved hydrophobic loop residues shown in blue and hydrogen bonds in red dashes. The 3_10_ helix in the connector linking the Vβ and Cβ domains is also observed in this region and shown in brown. TCR–pMHC binding is thought to engage the FG loop, which would then mechanically push against this 3_10_ helix and alter the overall TCR conformation. **(B)** Two views of CD3εγ are shown in which the linker region and several unstructured residues at the N termini of each domain have been omitted. CD3ε is depicted in blue and CD3γ in yellow. The structure below is rotated ~50° about the vertical axis relative to that on the top. The β strands are colored and labeled (blue for CD3ε and yellow for CD3γ). In the top structure, three pairs of main chain atoms involved in interdomain G strand hydrogen bonds are indicated with amide protons in gray and carbonyl oxygen atoms in red. In the bottom structure, the two pairs of disulfide-linked cysteine residues are shown as rods colored in magenta. Figure prepared using MOLMOL. **(C)** The heterodimeric CD3εγ subunit complex is illustrated, the CD3ε subunit is drawn in blue and the CD3γ subunit in yellow. Select interdomain hydrogen bonds are shown on the G strands as in **(C)**. The cytoplasmic tails are illustrated vertically to depict receptor length, whereas physiologically, the tails may be associated with the inner leaflet of the plasma membrane. Each CD3 subunit contains an extracellular, transmembrane, and cytoplasmic domain. The CD3εγ extracellular domain structure was generated from the deposited PDB file 1XMW using PYMOL. The inset shows a representative model of the CD3εγ CxxC region containing an intramolecular disulfide bond at the N-terminus of the TM helices. Only the cysteine residues C82 and C85 of CD3γ are shown for clarity. **(D)** Comparison of the membrane-proximal stalk length found in cell surface molecules present on the surface of T cells. Both the TCRα and TCRβ chains have unusually long stalks relative to other cell surface receptors.

The overall topology of the TCRαβ heterodimer is highly comparable to an immunoglobulin Fab fragment, as illustrated in Figure 2A. Each consists of a paired variable module (VαVβ vs. V_L_V_H_) joined to a constant domain module (CαCβ vs. C_L_C_H1_) and are of very similar heights and widths (16). However, upon closer inspection, marked differences are observed. First, the CαCβ module adopts an asymmetrical arrangement, resulting in exposure of residues on the Cβ domain ABD β sheet that are buried in the symmetrical Fab C_L_C_H1_ module (Figure 2A, green). Second, the breadth of the VβCβ interface differs from that of the V_H_C_H1_ interface. The buried surface area between the Vβ and Cβ domains on each side is ~350 Å^2^ in contrast to the ~150 Å^2^ between V_H_ and C_H1_. Almost one-third of the buried surface area in the VβCβ interface is due to the presence of a 12 residue long insertion referred to as CβFG loop (Figure 2A, yellow), which is unique to mammalian αβTCRs (18). As shown in Figure 2A right panel, the Cβ FG loop is highly structured. Centrally located in the FG loop is residue W223, in which two hydrogen bonds are formed from its indole NH group to the carbonyl oxygen atoms from Q225 and R227. Hence, the W223 residue is affixed in the middle of the loop to function as the crux of a mini-hydrophobic core that includes residues L217 and P230 (cyan color) (16). Moreover, a 3_10_ helix connector, exclusive to TCRβ, runs between the Vβ and Cβ domains in close proximity to this CβFG loop. A 3_10_ helix (brown) is a tightly wound secondary structural element and may extend in response to a force pulling on the segment, as is possible with the CβFG loop given the lack of disulfide bonds within the loop to prevent force-driven extension. Since removal of the CβFG loop-attenuated T cell function following antigen triggering (39, 40), we directed our efforts on this structurally unique feature of the αβTCR to assess if it is linked with mechanotransduction, as discussed below.

### CD3 heterodimers: CD3εγ and CD3εδ

The determination of solution NMR structures of CD3εγ (12) and CD3εδ (15), in addition to the crystal structures of antibody complexed CD3εδ (13) and CD3εγ (14), revealed key components of the αβTCR ectodomain architecture. Figure 2B illustrates the NMR structures of CD3εγ as an example. A summary of the most pronounced structural observations is as follows. The ectodomains of these CD3 subunits (as well as that of CD3δ, not shown) configure into a C-type Ig-like fold. Whereas CD3ε and CD3γ can be classified to the C2-set, CD3δ adopts the C1-set fold. Strikingly, the configuration of the CD3ε domain in structures of CD3εγ and CD3εδ are essentially identical, illuminating its structural fortitude. Of particular interest is the parallel association of the two CD3 heterodimers as illustrated for CD3εγ in Figure 2B (top). Extensive mainchain hydrogen bonds are formed between their respective G strands that enable the assembly of a conjoined “super” β sheet through the linkage of one ectodomain’s CFG sheet with another ectodomain’s GFC sheet, thereby shielding the hydrophobic residues in the interface.

This amalgamated β-sheet forms a highly stable yet squat ectodomain unit aligned vertically on the cellular membrane, well-constructed to facilitate T cell signal transduction events, as described below. The two CD3 heterodimers do display an obvious difference in geometry. A significant cleft between the ectodomain tops is readily observable in CD3εγ (Figure 2B, bottom); however, it is absent from CD3εδ (15). An analogous difference was also observed in the antibody bound human CD3εγ (14) and CD3εδ (13) crystal structures, affirming the significance of their topological distinctness across species. Notably, we have proven experimentally that this distinctly kinked conformation of the CD3γ G strand is critical for considerably augmenting antigen-triggered TCR activation and surface TCR expression (15). Moreover, this CD3εγ geometry accommodates the TCR β subunit’s juxtaposition, which is not possible in the case of CD3εδ (18).

### Intradomain disulfide bonds and CxxC connecting peptide motifs in CD3ε, CD3γ, and CD3δ

Another striking adaptation of CD3εγ and CD3εδ heterodimers is the “unorthodox” appearance of disulfide bonds both within and outside of the Ig-like ectodomains. Typically, these domains contain one disulfide bond formed between two cysteine residues, one centrally located in the B strand and a second in the F strand. As shown in Figure 2B (bottom), while these are conserved in both CD3 heterodimers, as exemplified for CD3εγ, in each domain the B strand cysteine is translocated to the top of the β strand. In effect, this rigidifies the “V” shape created by the upward pointing F strands. Thus, any pushing force on the CD3εγ ectodomains from above, including that via the Cβ FG loop, will readily be transferred to the superdomain and transmitted by the paired G strands to the TM domains.

A highly conserved CxxC motif between the junction of the extracellular and TM domains of each mammalian CD3ε, CD3γ, and CD3δ subunit has been previously determined to be crucial for thymocyte development and T cell activation (41–43). The redox state of each CxxC motif on resting and activated T cells was shown to be in a constitutively oxidized disulfide-bonded state using LC-MS and a biotin switch assay, consistent with measured reduction potential (44). A native oxidized CD3δ CxxC-containing segment was compared with that of a mutant SxxS-containing CD3δ segment in NMR chemical shift perturbation experiments. Extensive chemical shift changes were observed in residues within the membrane-proximal motif and additionally seen throughout the TM and cytoplasmic domains with removal of the native disulfide. Moreover, analysis of the native CD3δ segment in oxidizing and reducing conditions also reveals a multitude of spectral differences. These data show that the oxidized CxxC motif preserves the structural configuration within the membrane-proximal stalk region along with that of its adjoining TM and ITAM-containing cytoplasmic domains.

These results suggest that preservation of the CD3 CxxC oxidized state is essential for TCR mechanotransduction, thus bolstering a model in which the CD3 CxxC motifs rigidify the structure of the CD3 heterodimeric subunits within the extracellular to intracellular junction. The cysteines likely form a helical cap on the TM segment (Figure 2C, inset), with potential to mechanically couple the position and motion of the extracellular TCR complex to the intracellular signaling motifs. This rigidification adds to that of the squat ectodomains paired through extensive interface contacts and conjoint G strands (15). Martinez et al. (42) also propose that the CxxC motif of CD3ε may mediate allosteric regulation within a TCR oligomer. However, since the notion of TCR oligomerization is controversial and has recently been reviewed elsewhere [Ref. (45) and references therein], it shall not be discussed here.

Given that CD3 CxxC disulfides were resistant to reduction by the high concentrations of DTT present in the cellular experiments, beyond which cellular viability was compromised, it is unlikely that an environment exists in which these motifs are reduced at the surface of living cells *in vivo*. Consistent with this notion, cellular activation stimuli via TCR or CD2 pathways did not yield reduction (44), showing that CD3 CxxC motifs are not redox switches linked to T cell activation. The completeness of the oxidized state and the difficulty in reducing the CxxC motifs in a membrane-like environment may be a result of one or more factors. First, there may be a contribution to disulfide stability through the thermodynamic coupling of TM helix formation as has been reported in model studies on soluble helices (46, 47). Second, there may be some occlusion of the site due to its location within the CD3 molecule itself and/or due to ectodomain quaternary associations within the TCR complex (15). While this is not a factor in experiments studying the isolated CD3δ fragment, the position of the CxxC motif proximal to the central G strands of the CD3 heterodimers means that it is likely that significant steric hindrance would be encountered by an attacking thiolate anion, which must approach in line with the existing disulfide bond in order for thiol-disulfide exchange to occur. Similarly, the CxxC motif may be partially occluded by the lipid bilayer itself, with the TM helix retaining sufficient rigidity to prevent access by an incoming nucleophile to the disulfide bond. In sum, the CD3 CxxC motifs form highly stable intramolecular disulfide bonds on the surface of T cells that appear to be critical in maintaining the conformation and register of the CD3 subunits, TCR intersubunit interactions, and intracellular signaling responses to extracellular TCR–pMHC binding interactions.

The 5–10 amino acid short and rigid CD3 CP regions (41) are in sharp contrast with the more lengthy (19–26aa) and flexible TCRα and β CP regions, both of which link their respective constant domains to the TM segments. Note that the stalk lengths of the TCRα and TCRβ CP are two to three times greater than those of typical TM immunoglobulin receptors. This difference is still remarkable when the comparison is made counting just those residues outside of the Ig domain membrane-proximal region to the TCRα–β interchain disulfide (Figure 2D). Moreover, different lengths of TCR α and β CPs may cause an uneven distribution of mechanical load, possibly subjecting TCRβ to experience higher force with its shorter CP. This may also play a role during the development of thymocytes expressing a pre-TCR and thus lacking TCRα to those expressing TCRαβ heterodimers (see below).

## Vertebrate Evolution of the αβTCR Complex: Conjoint CD3 Molecular Speciation and CβFG Loop Evolvement

**Figure 3:**
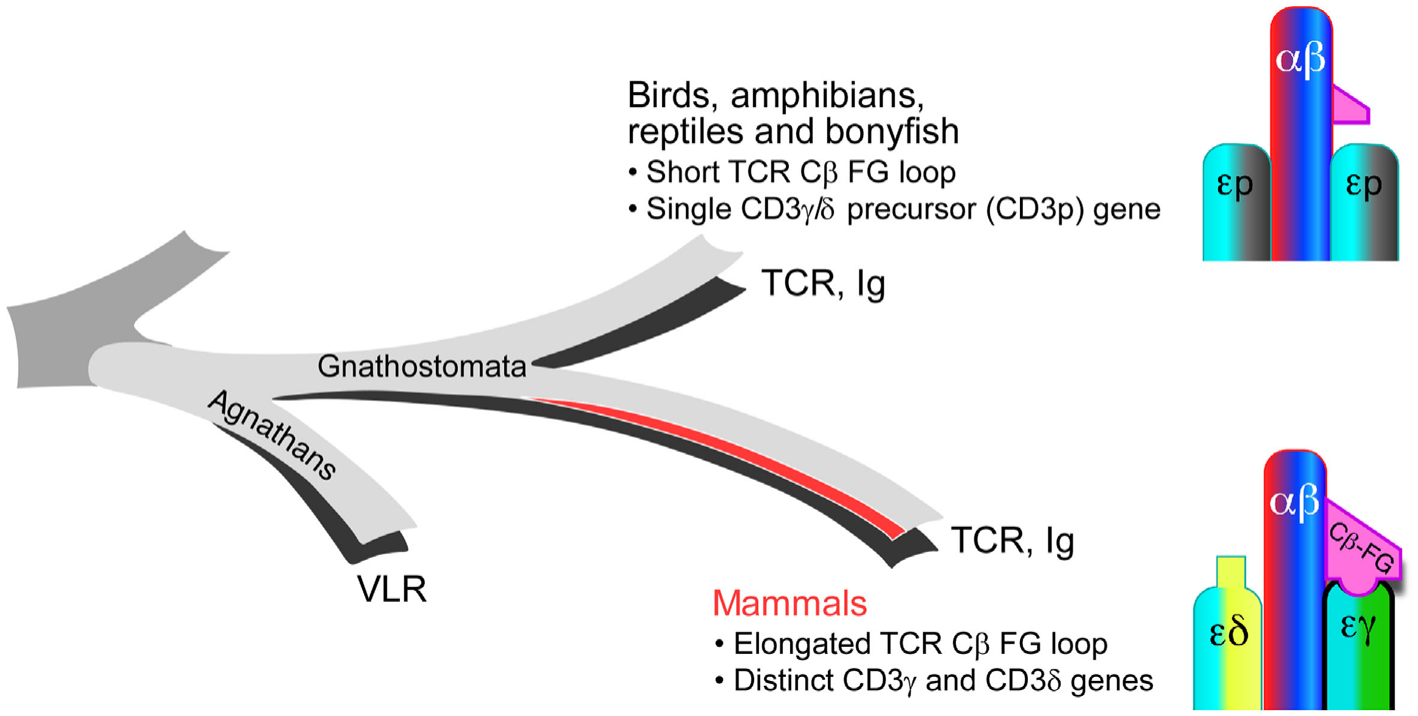
**Evolutionary coupling between the TCR Cβ and CD3 gene products**. In mammals, the TCR CβFG loop is significantly elongated and distinct CD3γ and CD3δ genes are present relative to the TCR genes found in birds, amphibians, reptiles, and bony fish. These mammalian adaptations during molecular speciation likely establish a more advanced immune system with greater sensitivity for pMHC recognition.

Whereas the striking elongation of the CβFG loop among mammalian species is well conserved, sequence comparison with non-mammalian vertebrate species (chicken, fish, and frog) reveals that the lengthy CβFG loop is not observed in the latter (18). The absence of distinct CD3γ and CD3δ subunits in the above-mentioned non-mammalian species and expression of a single precursor CD3γδ gene (CD3p) have been shown based on the genomic and biochemical analyses as well as theoretical predictions dating the required CD3 duplication event (48–50). Although recently, it has been revealed that the jawless vertebrates (agnathans) have an alternative adaptive immune system with variable lymphocyte receptors (VLR), all jawed vertebrates (gnathostomata) possess a fully developed adaptive immune system with TCR and Ig genes (51). Both the elongated CβFG loop and the distinct CD3γ and CD3δ genes are unique in the mammalian species among gnathostamata (Figure 3). These analyses support the notion that TCRβ and CD3γ have been evolutionarily coupled for TCR assembly and signaling in the mammalian species. Furthermore, these findings imply that the distinct topology of CD3 heterodimers co-evolved with TCR Cβ domains to optimize the quaternary TCR structure for pMHC-triggered αβTCR activation. TCRαβ heterodimer assembly studies with various CD3 complexes, using a phylogenetic approach support this conclusion (52).

The recent NMR structure of chicken CD3εp reveals a unique dimer interface with surface exposed, non-conserved residues clustered to a single face of the heterodimer (53). These, among other details, suggest that the orientation of the two CD3εp heterodimers in a given TCR complex in non-mammals is similar to that shown in Figure 1 for the mammalian counterparts.

## TCR Quaternary Change upon pMHC Ligation

**Figure 4:**
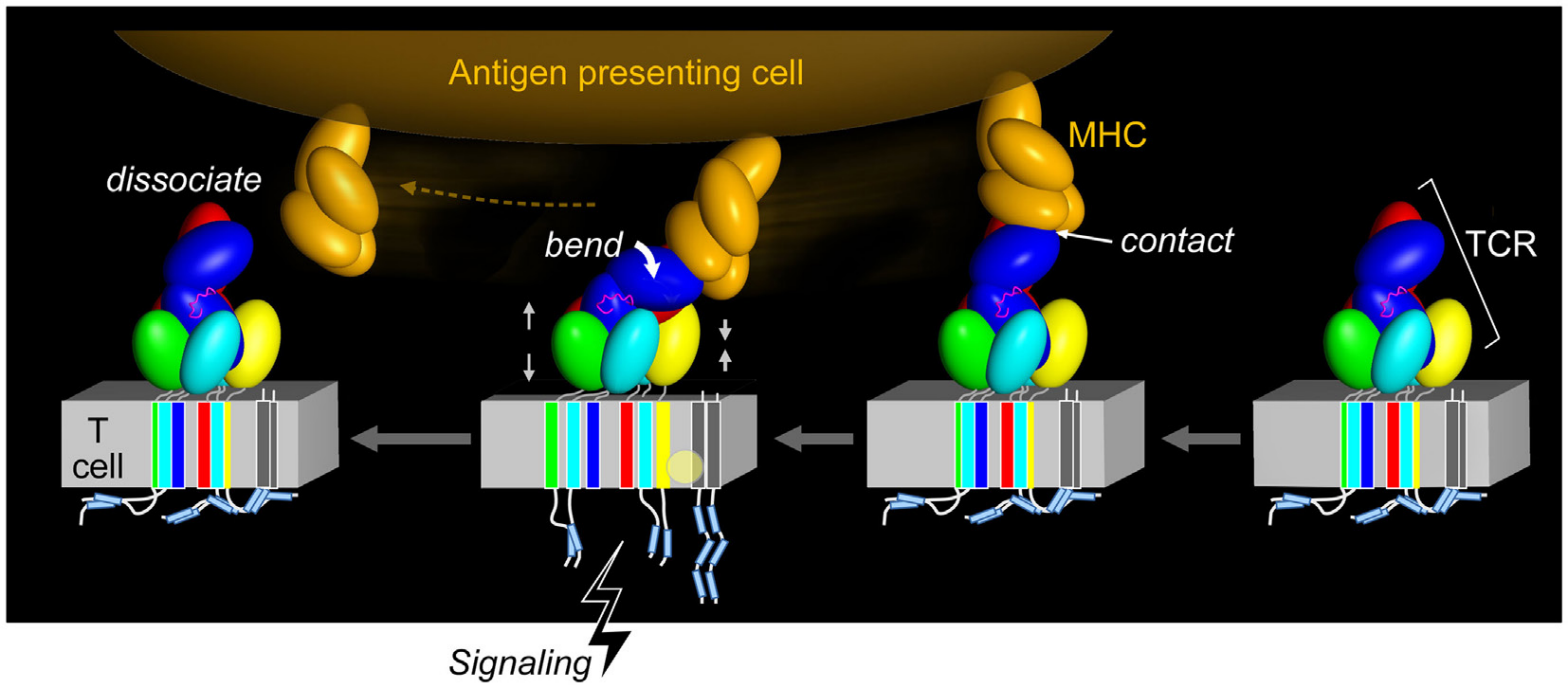
**Force initiated TCR signaling**. The force generated from the pMHC interaction with the TCR results in allosteric changes within the TCR subunits, most notably in the positioning of the TCR CβFG loop and rearrangement, and impacts the VαVβ module and quaternary αβTCR subunit changes. This restructuring through compression and tension forces (denoted by gray facing or opposing pairs of arrows) may alter the arrangement of the TCR TM domains and co-occur with changes in the membrane lipid composition. The ITAMs are thought to be released from the plasma membrane and become available to tyrosine kinase phosphorylation, thereby initiating T cell activation. Upon TCR–pMHC dissociation, the TCR returns to its initial disengaged state. In the illustration, the CD3ζ cytoplasmic tails are shown to be membrane associated, where the ITAM tyrosine residues are embedded into the membrane and consequently shielded from phosphorylation, as has been observed for the CD3ε cytoplasmic tail (54–58). Other studies have demonstrated that the CD3ζ subunits exist in a constitutively phosphorylated state and therefore would not be associated with the lipid membrane as depicted (59–61) (pMHC, orange; CβFG loop, magenta; TCR complex, other colors; lipid alteration, yellow circle).

The functional importance of this contrasting arrangement of squat and rigid CD3 CP segments with respect to flexible TCR α and β CP was revealed when interactions of activating (i.e., 2C11 or 500A2) and non-activating (17A2) anti-CD3ε mAbs were compared. These antibodies bind the CD3εγ ectodomains with virtually identical affinity on T cells (17), but the activating antibodies bind the membrane-distal CD3ε lobe, approaching diagonally and adjacent to the CβFG loop, a structural feature previously noted to facilitate pMHC-triggered activation (40). By contrast, the non-activating mAb 17A2 binds between CD3ε and γ perpendicular to the T cell membrane (17). Only the application of force tangential to the T cell surface allowed bead-bound 17A2 mAb to become stimulatory as measured by an increase in calcium flux. Specific, but not irrelevant, pMHC also activates T cells only when pN-scale tangential mechanical force is applied via optical tweezers. During immune surveillance, these findings suggest that the TCR acts as a mechanosensor, using the movement of the T cell relative to the APC, to provide mechanical force to initiate signaling cascades. This paradigm is illustrated in Figure 4 wherein TCR–pMHC recognition occurs, immediately pulling on the TCRαβ heterodimer as the T cell moves in apposition to the APC. This force is transferred through the CβFG loop to CD3εγ with the horizontal force converted to vertical force in a lever-like motion with the TM of TCRβ acting as a fulcrum. Signaling is initiated rapidly, before the integrin-mediated stop signal occurs. Only tangential force activates the TCR, meaning that it is an anisotropic mechanosensor (17). The lateral pull from pMHC, exerting compressive and tensile stresses, relays force through the CβFG loop, focusing the push on the upper outer surface of CD3ε. Given the diversity of TCRs and the variety of docking modes with their pMHC ligands, it is likely that a concerted quaternary motion together with conserved structural transitions described below can reliably initiate signals. Glycans, acting perhaps as springs, may nuance these mechanisms by serving as energetic barriers to signaling via CD3 subunits (33). Data described in the section to follow show how force structurally alters the TCRαβ heterodimer itself.

Figure 4 also suggests that force causes conformational change within the TM segment assembly of the αβTCR. In turn, this facilitates exposure of the ITAMs for lck-mediated phosphorylation of the CD3 cytoplasmic tails and induces lipid rearrangements in the receptor-proximal area that may be critical for signaling. Subsequent dissociation of pMHC from the αβ TCR may revert some or all of these changes. However, in the context of the immunological synapse and continued triggering by pMHC, cytoskeletal attachments promulgated by signalsome-related scaffolding and the like may sustain the predicted conformational changes by providing inside-out force.

## TCRαβ Structural Transition and Allosteric Regulation of Peptide Discrimination and pMHC Bond Lifetime

**Figure 5:**
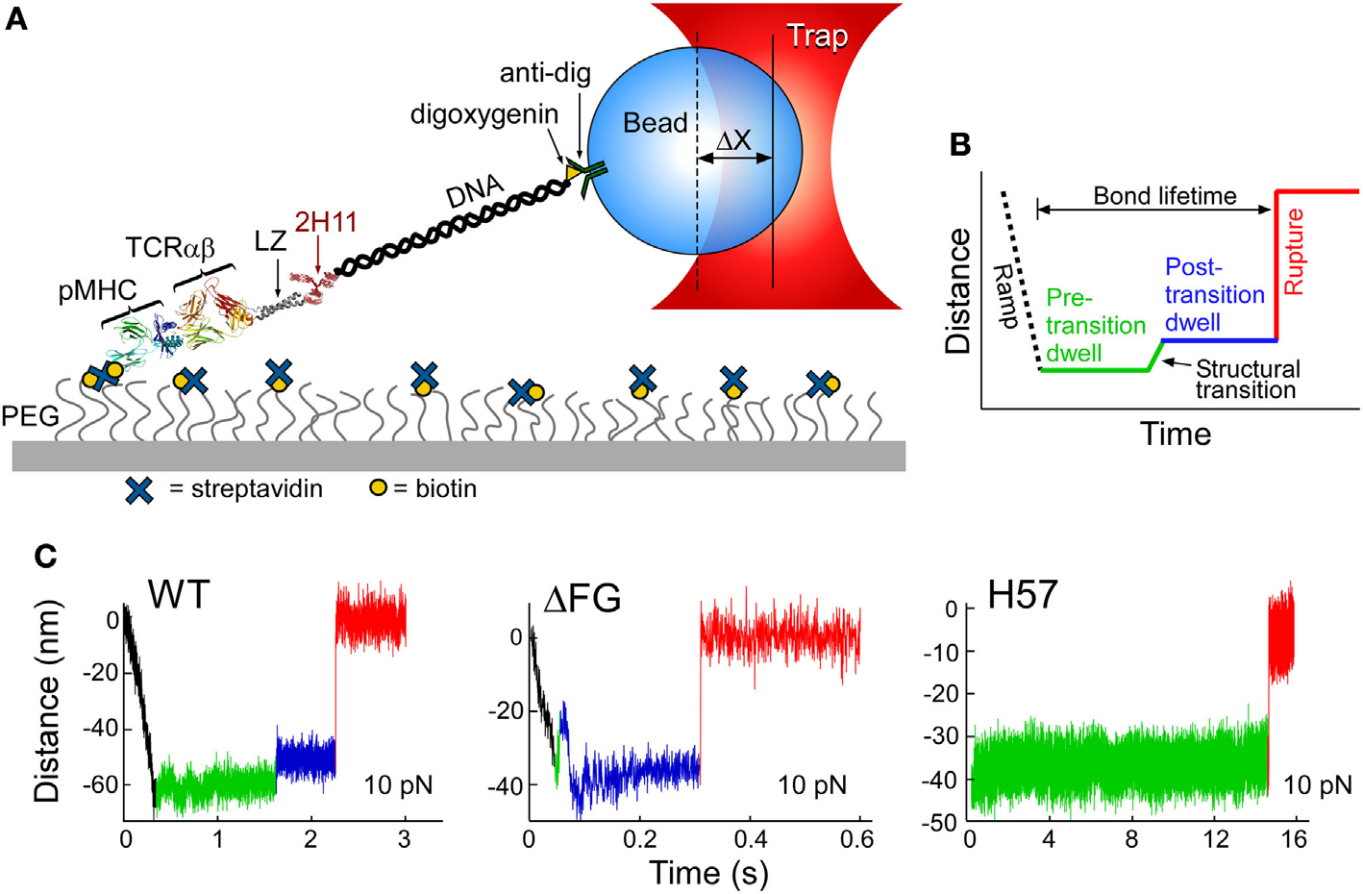
**Single-molecule TCRαβ–pMHC studies identify structural transitions**. **(A)** The lifetime of αβTCR–pMHC bonds are analyzed by the single-molecule tether assay shown. “ΔX” represents the displacement of bead from the trap center. **(B)** Loading profile for measuring bond lifetime. Larger separation along the system path is shown through an increase in distance. A black dashed line represents an initial ramp phase, during this stage the tether is loaded to a fixed force and a green line illustrates the “Pre-transition dwell.” A signature structural transition is observed repeatedly, indicated by a green to blue line, and then followed by a “Post-transition dwell,” in blue. “Rupture,” shown by red line, is observed as an abrupt upward step. **(C)** Representative traces for VSV8/K^b^ at 10 pN for WT, ΔFG, and H57 Fab-bound TCRαβ. A commonly occurring transition and rupture is seen in the WT trace. An early transition is often detected for ΔFG, here occurring amid the initial ramp phase. H57 Fab traces do not show a transition nor a dwell for longer periods before rupture. The green baseline (see WT) illustrates observed low-amplitude motions.

The TCRαβ–pMHC complex has recently been found to undergo significant transitions under force load (62). By isolating a TCRαβ–pMHC interaction to a coverslip surface and tethered bead (Figure 5A), force was applied to the complex via an optical trap. By translating the sample relative to the fixed trap and holding it at a fixed position, and thus force, until bond rupture, bond lifetimes could be measured. Upward displacements of the bead within the trap (Figures 5B,C) were interpreted as conformational extensions. Conformational transitions spanning 8–15 nm (Figure 5C, green to blue) were frequently observed, representing an extension of the system by domain rotation, extensions, and/or unfolding. The CβFG loop is strongly implicated in these transitions since removing the CβFG loop causes TCRαβΔFG transitions to occur much earlier in the pulling experiment (compare Figure 5C, wt vs. ΔFG). Additionally the CβFG loop-binding H57 Fab (16), essentially abolished any transition (Figure 5C H57), presumably stabilizing this region against structural unfolding. Lastly, a mutant TCR in which the FG loop is deleted (ΔFG) (39, 40) increased the magnitude of displacement, as described below.

**Figure 6:**
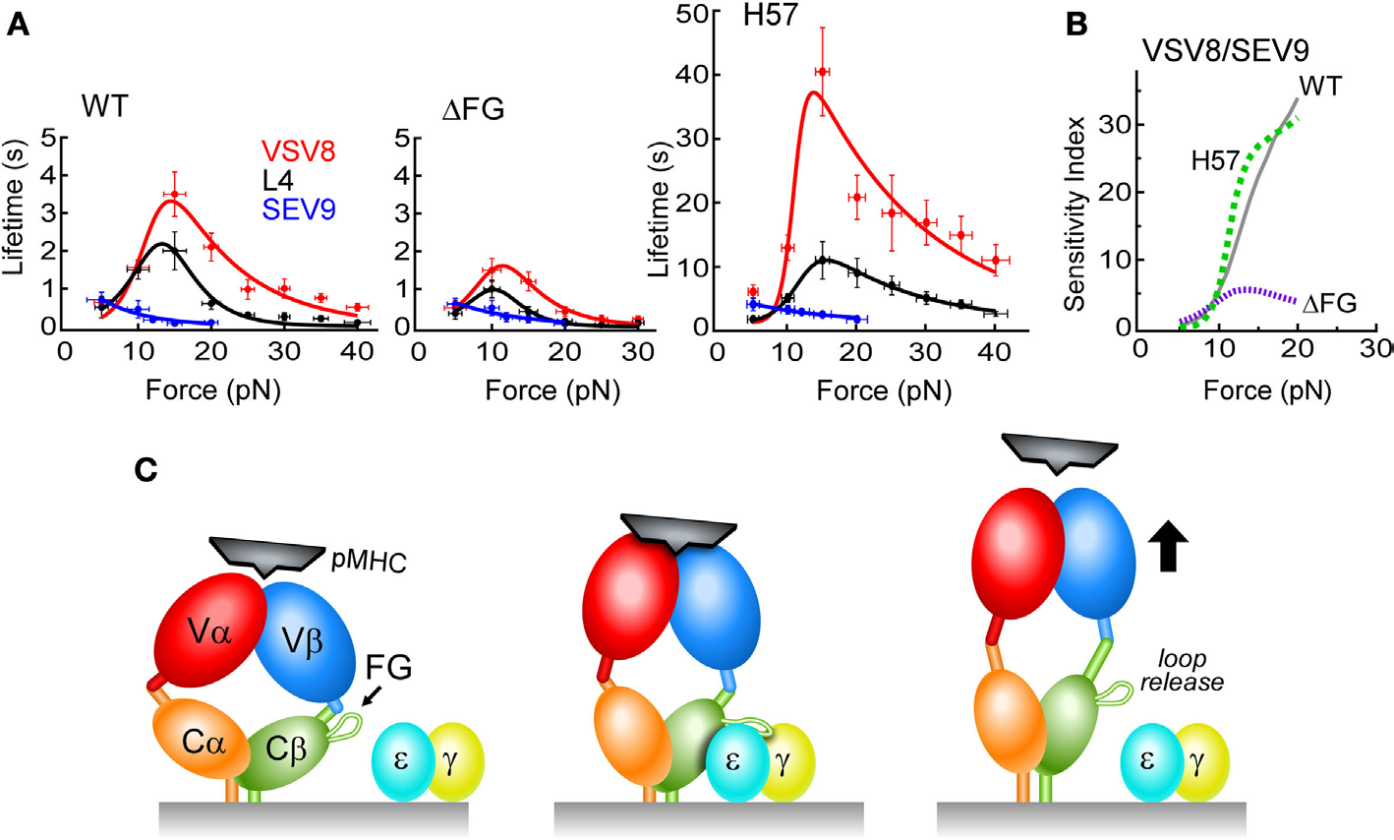
**N15 TCRαβ–pMHC force/lifetime curves, sensitivity indices and schematic diagram of low affinity and high affinity TCR binding mechanisms**. **(A)** Force-bond lifetime plots for TCRβ WT, ΔFG, and H57 Fab-bound N15αβ. Catch bonds are observed for both VSV8 and L4 whereas slip bond character is seen for SEV9. Catch bonds peak at ~15 pN for WT and shift to lower force for ΔFG with significant reduction in bond lifetimes. Dramatic increases of catch bond lifetimes occur as a result of stabilization of the CβFG loop by the H57 Fab. **(B)** Sensitivity plots comparing TCRαβ–pMHC bond lifetime ratios of VSV8/SEV9 antigen for WT (solid, gray), H57 Fab (dashed, green), and ΔFG (dotted, purple). **(C)** Left, the TCR (multicolored) is in an unloaded, compact state with weak pMHC binding affinity depicted as loosely fitting pMHC (black). Subtle conformational motions may be occurring while gating potential interactions. Center, the TCR is in a loaded and more extended state, possessing high binding affinity resulting from significant structural rearrangements that create catch bonds. The pMHC is tightly bound to the TCR, resulting in the force generating engagement of the CβFG loop in a conformation binding the CD3εγ cleft for association (CD3ε, blue; CD3γ, yellow). Right, the TCR adopts a fully extended conformation where one or more conformational changes occur: CβFG loop disengagement, angular changes between the V to C domains and a weakened TCR–pMHC binding interface. For clarity, the CD3εδ heterodimer is not depicted.

Bond lifetimes as a function of force were found to vary with the pMHC ligand, suggesting a force-dependent mechanism for peptide discrimination (Figure 6A). H-2K^b^, a class I molecule, was complexed with a strong agonist (VSV8), a weak agonist (L4), or the Sendai virus-derived SEV9, which binds to K^b^ comparably to VSV8 but does not activate N15-expressing T cells (63). There was found to be a catch bond with peak lifetime at ~15 pN for VSV8, a weaker catch bond with shorter lifetimes for L4 and only slip bonds for SEV9 (Figure 6A). With ΔFG, there was shortened bond lifetime, with maximal lifetime shifted to ~10 pN (Figure 6A) and lessened ability of the TCR to discriminate different ligands (see below). By stabilizing the CβFG loop via H57 Fab, catch bond lifetimes were increased significantly (Figure 6A). By contrast, the Cα binding antibody H28 only modestly extended bond lifetime (not shown). Single-molecule measurements on isolated single cells (SMSC) corroborated the SM observations and showed that catch bond regulation was not dependent on the CD8αβ co-receptor (62). Moreover, the early transition manifest by N15αβΔFG correlated with a 100-fold decrease in sensitivity to VSV8 peptide as judged by IL-2 assay.

In short, strong evidence for allostery was revealed, correlating the state of the CβFG loop to the strength of the TCRαβ–pMHC bond. Moreover, a greater mechanical extension was determined to be linked to ligand potency and CβFG loop region structure (62). A model was formulated in which an unloaded compact TCR binds weakly, a loaded compact TCR binds strongly, with binding strengthened by stabilizing the CβFG loop region. Release of ligand would then occur through an extended TCRαβ (Figure 6C). In intact single cell experiments where the entire αβTCR complex is surface expressed, adjacent CD3εγ ectodomains may stabilize the CβFG loop region, prolonging bond lifetime, and transmitting force important for signaling. That TCR–pMHC bond lifetime is increased by H57 binding (Figure 6A) but pMHC-triggered T cell responses are blocked (17) is consistent with the notion that bond lifetime is necessary but not sufficient for signaling. Our model posits that the CβFG loop enhances mechanosensor function through force-activated gating of initiation and stabilization of productive pMHC interactions with release of unproductive interactions, thereby controlling catch bond strength, bond lifetime, and CD3-dependent activation (Figure 6C).

A differential of ~40 Å in transition is observed (Figure 5C) between agonist relative to irrelevant pMHC ligation of the N15 TCRαβ (62). Simple extension of the Vβ–Cβ connector would only permit about a 15 Å transition and indeed, displacements for TCRαβΔFG were greater than wtTCRαβ (62). Therefore, one must look outside of the FG loop for the source of these TCRαβ heterodimer-pMHC rearrangements. Possible sources may include extending the long CPs between Cβ and the LZ TM surrogate used in the assay constructs and Cα and LZ. The Vβ and Vα domains may possibly slide relative to one another at their hydrophobic interface. While major motion within individual V and C domains of the TCR heterodimer would be restricted by conserved intradomain disulfide bonds in each, the A and/or G strands could be pulled free with force. Reversible low-amplitude 1–3 nm movements of the baseline (see Figure 5C, left panel, for example) also occurred, suggesting smaller reversible reconfigurations within the αβTCR–pMHC complex.

Modes of CD3εγ association within pre-transition, intermediate or post-transition states are not clear. Binding of CD3 to pre-transition or other compact states might function similarly to H57 Fab by extending the lifetime of the αβTCR–pMHC bond. The bound CD3 heterodimer may participate in the transitional mechanical change, being pushed and pulled to expose membrane or protein sequestered ITAMs within CD3 cytoplasmic tails. Post-transition binding to extended TCR states may also modulate CD3 binding in a currently undefined positive or negative manner.

## Dynamic Bond Formation: Linkage to Kinetic Proofreading

Our findings address how αβTCRs, with extremely weak solution affinities, can mediate the biologically observed sensitivity and specificity. They are able to differentially detect a few peptide side chains in the context of the MHC groove despite sharing the majority of recognition surface contacts with other pMHC complexes. Many identically sized peptides are bound to the same type of MHC molecule on the surface of an APC, forming an array of ~100,000 different pMHC complexes. The T cell must use its ~ 20,000 αβTCR surface receptors to scan this diverse collection of pMHCs rapidly and accurately to find a dozen or fewer relevant foreign peptides. We posit that load-induced transitions tune αβTCR–pMHC bond lifetime; maximal non-linear dynamic bond lifetime is associated with TCRαβ structural transitions distinguishing readily among peptides, detecting differences of only a single residue (VSV8 vs. L4). In the absence of the Cβ FG loop driven allostery or force, discrimination is minimal (Figure 6B). As an αβTCR is gaffed (i.e., caught) by its specific pMHC, force is generated, bond lifetime extends, and a structural transition is induced. Dynamic bond formation and its associated transitions likely are sufficient to promulgate αβTCR quaternary change, CD3 heterodimer interactions, and TM and cytoplasmic tail segment alterations leading to signaling downstream (6, 17, 64). A very few productive interactions will result in T cell activation as a quantal phenomenon that will be sustained further at the immunological synapse (3, 65). Unrelated or variant peptides will not induce TCR transitions or extension of bond lifetime sufficiently (Figure 6) to initiate a signaling event.

The mechanical transition that prolongs the αβTCR–pMHC bond lifetime has a number of relevant consequences. First, this extension highlights a previously unknown state. As seen in integrin αβ heterodimeric systems, affinities can vary greatly between compact and extended states. The αβTCR appears to work with an opposite logic to that of the integrins; the compact form binds strongly while the extended form does so weakly. In integrins with plentiful ligands, the “off state” is the default starting state, while for the αβTCR the “on state” is the initial state, since the αβTCR must seek out rare binding events. The second consequence is that ligand-dependent displacement seen here may differentially couple to mechanisms that critically depend on distance, such as TCR–APC intercellular membrane proximity. Third, the CβFG loop region controls the mechanical transition in the αβTCR–pMHC likely occurring through the physiological CD3 associations noted above. Thus, the constant region of the TCR controls the mechanism of discrimination, allowing near-infinite variability in the ligand-recognition elements while preserving the essential function of the TCR. Lastly, to distinguish among highly similar ligands, kinetic proofreading mechanisms are generally energy intensive, requiring a system to work outside of thermal equilibrium (64, 66). Mechanical work can drive non-equilibrium signaling events. Triggering is sustained during immune surveillance and at the immunological synapse through the use of forces derived from coincident cell movements. Although many models of T cell function in antigen recognition have been suggested [reviewed in Ref. (64)], the mechanosensor concept is the most compelling in our view.

## The Pre-TCR is a Self-pMHC Mechanosensor

β selection is the first major checkpoint in early thymic development. Signaling through the pre-TCR terminates β gene rearrangements and rescues DN thymocytes from apoptosis while inducing massive expansion of thymocytes (67). Subsequently, the pre-TCR promotes the differentiation of DN thymocytes to DP thymocytes, triggering TCRα gene rearrangements and generating millions of αβTCRs with distinct pMHC specificities via their paired Vα and Vβ domains [Ref. (68) and references therein]. Earlier work (69) suggested that pre-TCR signals autonomously. Based on X-ray crystallographic data, it was further suggested that the pre-TCR might form a superdomain of two pre-TCRs positioned in an anti-parallel fashion proximal to the membrane, activating in the absence of ligand binding (70). Rather, it is possible that a Vβ domain that is not paired with Vα can extend from the thymocyte membrane in an upright orientation as a monomer or dimer, in a fashion more similar to TCRαβ, providing an alternative model of pre-TCR conformation (71).

Camelids possess a fully functional class of antibodies devoid of light chain (72) while antibody V_H_ domains in mammals are often major determinants of antigen affinity and specificity (73). Concordantly, endogenous retroviruses and bacterial superantigens interact strongly with mature αβTCR Vβ domains (74). Moreover, it was apparent from these crystallographic analyses that the CDR loops in the canonical ligand binding region were arranged in a manner similar to that found in the TCRαβ (70, 71), suggesting ligand competence. Together with a hydrophobic patch within Vβ, the CDR loops could combine to create a surface of considerable dimensions. We reasoned thus that the Vβ could be capable of interacting with pMHC ligands. Utilizing NMR and single-molecule BFP studies, we found that β subunit binds pMHC utilizing Vβ CDRs and the Vβ patch region (34). Force was found to regulate single pre-TCR–pMHC bonds similarly to the αβTCR with respect to TCRαβ–pMHC bonds (38), but with more promiscuous ligand specificity. Importantly, ligand interaction was found to robustly trigger calcium flux. Thus, the β repertoire is tuned prior to the αβ repertoire tuning, with pre-TCR interactions with self-pMHC modulating early thymocyte expansion. This has significant implications for β selection, immunodominant peptide recognition and the germline-encoded MHC interaction, suggesting that some process within β must be optimized prior to the αβTCR stage to tune maximum fitness for foreign peptide/self-MHC recognition by αβTCR on mature T lymphocytes.

In fact, it seems likely that pre-TCR–self-pMHC interaction is a key basis to expanding DN3 thymocytes and facilitating progression to the DP stage where αβTCRs are expressed. Thus, DN progression selects for a self-reactive repertoire early in development. The Vβ patch may contribute to this behavior, relaxing peptide specificity requirements and functioning as a surrogate Vα domain whose replacement at the DP stage by an actual Vα domain then imposes more precise peptide recognition. Negative selection therein purges high pMHC self-reactivity while maintaining a low self-pMHC bias. Given the presence of the FG loop in the pre-TCR, it is likely that the CβFG therein also exerts allosteric control over bond lifetime through force-dependent conformational change. It may be the case that the DN3–DP transition checkpoint ensures that VDJ recombination does not introduce CDR3 structural elements incompatible with mechanotransduction, thereby breaking the apparatus. Molecular details of the role of the Vβ hydrophobic patch fostering a tilted ligand binding geometry relative to αβTCR will illuminate how force-transduction through the β subunit diverges relative to the mature αβTCR (34). In the pre-TCR, given these interfacial and geometric shifts, the unpaired Vβ domain should certainly behave differently under force in comparison to when it lies in the context of the paired TCRαβ V module.

## Future Direction

It has been several years since the notion of TCR mechanobiology was reviewed in this journal (33). At that time, preliminary data were discussed on TCR topology and the striking requirement for tangential force applied along the pseudo twofold symmetry axis of the TCR complex by pMHC essential for T cell activation. pMHC ligation *per se* was not sufficient. The potential for non-equilibrium catch bond formation important for cognate recognition was suggested.

Since that time, catch bonds have been directly observed and elegantly studied by Zhu and colleagues in the OT1 system (38). It was observed that the TCR forms a dynamic bond, in which the bond lifetime increases with increasing force, when presented with an agonist peptide. The strength of the agonist is directly related to the duration of the bond lifetime; the stronger the agonist, the longer the bond lifetime. The positive correlation of agonist peptide activity to bond lifetime is in contrast to what is observed when antagonist pMHC complexes associate with the TCR. Antagonist binding results in slip bond formation, in which the bond lifetime decreases with increasing force. The activity of the peptide is also coupled with the cumulative lifetime of the TCR–pMHC bond. Recent single-molecule assay development and molecular cloning techniques have allowed us to develop methods to monitor nanometer extensions and piconewton forces to probe TCR–pMHC bonds under load (62).

By creating mutant β chain molecules to study behavior of the FG loop structure *in vitro* under load as well as *in vivo*, relevant TCRαβ–pMHC and pre-TCR–pMHC interactions can be investigated. We hypothesize that the CβFG loop modulates the strength of the TCR–pMHC bond by serving as a mechanical controller. When unloaded, it “gates” the binding and when loaded, it either stabilizes the initial compact conformation by lengthening of the catch bond lifetime for signaling through CD3εγ, or directs the system through a release pathway by controlling the Cβ–Vβ connector, which in turn extends and lets go of pMHC (Figure 6C). Hence, mutations that stiffen and even lock in, or conversely, loosen this “gate” will alter the mechanobiology vis-à-vis catch bonds, mechanical displacements, etc., and should perturb T lineage function in immature thymocytes as well as mature T cells.

The conformational motion of domains under load can be studied by single-molecule fluorescence. Direct observation of mechanical displacement or visualization through resonance energy transfer (FRET) studies and *in silico* modeling/simulations of structural arrangements can be analyzed using these fluorescence measurements. In this way, the origin of conformational transitions in mechanosensing can be studied. All-atom molecular dynamic simulations (75–77) of the TCR and pre-TCR can be used to calculate conformation-dependent forces (78), which will allow us to gain an atomistic view of the role of mechanical load in changing quaternary structure. We predict that the load-induced changes of the TCR–pMHC interface primarily involve rearrangements where new hydrophobic contacts form due to their isotropic nature compared to more directional hydrogen bonds. These structural changes shall correspond to the observed catch bonds in our view.

With respect to analysis of the critical importance of CP and CxxC motifs, in particular, SCSM assays shall be invaluable. For example, since the CD3 CxxC motif performs a pivotal role in extending the structural rigidity of the paired CD3 heterodimers and their respective TM helices, one would predict that loss of the CxxC motif through mutagenesis should reduce the force activation mechanism of the T cell. In essence, this would create a loss of binding energy and disrupt mechanical coupling to and within the CD3 heterodimers due to increased flexibility. Thus, more force would be required to activate T cells with such mutations. Structural studies can further reveal interactions among CPs, as between TCRα and CD3δ or any other segments. In conjunction with nanodisc technology and high resolution cryoEM, αβTCR complex structures should be possible to obtain. Collectively, these studies will afford unprecedented, detailed insights into the immunobiology of cognate T cell recognition. Its relevance should be evident at both basic and translational levels.

## Conflict of Interest Statement

The authors declare that the research was conducted in the absence of any commercial or financial relationships that could be construed as a potential conflict of interest.
